# A randomized clinical trial on innovative functional esthetic appliance for craniofacial growth modulation with 3D analysis of TMJ

**DOI:** 10.34172/joddd.025.41487

**Published:** 2025-03-31

**Authors:** Shubhangi Mani, Nandlal Girijalal Toshniwal, Yash Goenka, Nikita Navgire, Ankita Khurdal

**Affiliations:** Department of Orthodontics and Dentofacial Orthopaedics, Rural Dental College, Pravara Institute of Medical Sciences (DU), Loni, Maharashtra, India

**Keywords:** Cl II malocclusion, Clear functional jaw corrector, Dental changes, Skeletal changes, Twin block appliance

## Abstract

**Background.:**

The present study evaluated condylar position changes using cone beam computed tomography (CBCT) in treating Cl II malocclusion with the twin block and clear functional jaw corrector (CFJC) appliances.

**Methods.:**

In this RCT, we included 60 patients, with 30 in each treatment group (control group: twin block appliance, case group: CFJC appliance), randomly allocated using a lottery system. A twin block appliance or CFJC was fabricated for each patient following the protocol. Pre- and post-treatment records were collected over twelve months at 0-, 6- and 12-month intervals using cephalograms, CBCT, and questionnaires assessing the patient perception of the appliance.

**Results.:**

Both groups showed significant improvements in malocclusion. Cephalometric analysis showed statistically significant differences between the two groups in SNB, ANB, and U1-NA. In comparing the two groups, significant differences were found in Arnett’s soft tissue parameters, including upper lip to E line, lower lip to E line, upper lip protrusion, upper lip length, lower lip length, lower 1/3 of the face, maxillary first incisor exposure, and mandibular height in the CFJC group. The intergroup comparison of projections to TVL (true vertical line) also showed significant differences across all parameters in the CFJC group. Furthermore, significant disparities in CBCT parameters were observed between the groups, specifically in condylar position, condylar height, and anterior joint space. Also, significant differences in patient comfort and perception of the appliance were observed, highlighting better compliance with the CFJC appliance.

**Conclusion.:**

The CFJC appliance is a top choice for Cl II malocclusion due to its superior effectiveness in skeletal, dental, and soft tissue improvements and significant condylar remodeling. Additionally, patients showed better compliance and acceptance of the CFJC appliance compared to traditional options, enhancing its clinical advantage in orthodontic practice.

## Introduction

 The orofacial region is vital for interpersonal interactions and communication. Malocclusion, a deviation from normal tooth alignment, often requires orthodontic treatment to achieve optimal occlusion, balancing function, stability, and aesthetics. Cl II malocclusion, affecting about one-third of the population, is typically characterized by mandibular skeletal retrusion and can impact respiratory function and sleep. ‘Airway friendly orthodontics’ involves functional therapy to enhance mandibular growth.^1^ Various removable functional appliances, such as the activator, bionator, Frankel, and twin block, are used to correct Cl II disharmony. The twin block, developed by William J. Clark, is particularly popular for its effective, speech-friendly design.

 Understanding the twin block mechanism is crucial for orthodontists. This appliance primarily induces sagittal changes, increasing mandibular length and improving the facial profile from convex to straight. It enhances the anteroposterior diameter and condyle height, repositions the condyle forward, and causes backward disk movement. Modifications in the glenoid fossa due to tissue stretch and altered synovium flow result in significant bone formation within six months. These changes, influenced by viscoelastic tissues, occur alongside skeletal, dental, neuromuscular, and age factors. However, functional appliances can cause discomfort, including mucosal pressure, soft tissue tension, and speech difficulties, impacting patient compliance.^2^ Orthodontists must select suitable appliances and manage discomfort effectively.

 Patient compliance significantly influences the success of removable orthodontic appliances.^3^ O’Brien et al^5^ noted that non-compliance often hampers early twin block treatment.^4^ The bulkiness and visible wires of traditional appliances contribute to non-compliance. Newer, more comfortable, and lightweight wireless appliances are needed. Though effective with better compliance, the fixed twin block can cause gingival inflammation, food lodgment, foul odor, and discomfort.^6^ Clear aligners, offering better esthetics and comfort, show promise. They reduce gag reflexes and improve patient satisfaction. The “clear twin block” developed by Behroozian and Klaman retains traditional benefits while enhancing comfort and esthetics by eliminating wire elements, increasing patient compliance and treatment efficacy.^7^ Recently, there has been a transition from traditional braces to transparent tooth positioners or aligners for treating mild to severe crowding and extraction cases. As a result of the growing demand for esthetic solutions, the clear mandibular advancement appliance was developed. This appliance combines features of both a functional appliance and an aligner, offering an alternative treatment option.^8^

 Many studies have evaluated the skeletal outcome of twin-block treatment with mixed findings. Some reports have shown significant mandibular growth,^9^ while others note primarily dentoalveolar changes.^10^ Duan et al^11^ found that twin-block appliances effectively reduced pediatric OSA symptoms. Mandibular growth is linked to temporomandibular joint (TMJ) responses, and studies using CBCT have shown forward condylar positioning and remodeling.^12^ Effective TMJ changes involve condylar changes, glenoid fossa remodeling, and condylar displacement. There is a lack of studies with larger sample sizes, comprehensive evaluations of skeletal, soft tissue, and TMJ changes, along with compliance factors.^13-18^ Therefore, we conducted this study to obtain more conclusive results.

## Methods

###  Fabrication of prototype

 Thermoformed vacuum clear “copyplast” material of various thicknesses was used to fabricate prototypes for the clear functional jaw corrector (CFJC). Acrylic blocks with various mechanical adhesive mechanisms were incorporated into these prototypes. After testing different thicknesses, a 1-mm thickness of the clear thermoformed sheets was chosen for its durability and ability to withstand mechanical forces. Small grooves along the sides of the acrylic blocks were added, which proved effective in retaining the blocks securely within the CFJC appliance. This design ensured both functionality and durability, making it suitable for clinical use.

###  Clinical study

 The present experimental study was performed in 2 years (May 2, 2022, to April 30, 2024) with an observational period of one year. The study was approved by IEC and conducted in the Orthodontics and Dentofacial Orthopaedics Division, Rural Dental College, Loni.

 Outpatients from the department were selected based on eligibility criteria. The total sample included 60 subjects determined using Dr. Kulkarni’s software comprising both sexes randomly divided into two groups by lottery method:

 T: The test group using a CFJC

 C: The control group using a twin block

###  Inclusion criteria

(i) Age group of 12‒16 years with CVMI stage 3‒4 and MP3 stage F to FG eligible for myofunctional therapy
(ii) Skeletal Class II with orthognathic maxilla and retrognathic mandible (Salzmann Class II type 2) eligible for myofunctional therapy
(iii) Angles Class II div 1 malocclusion with increased overjet and overbite eligible for myofunctional therapy
(iv) Patients with early permanent dentition eligible for myofunctional therapy
(v) Patients with well-aligned dental arches eligible for myofunctional therapy
(vi) Patients ready to give written informed consent and participate in the clinical trial, who were eligible for myofunctional therapy

###  Exclusion criteria

(i) Patients with numerous local/systemic problems/syndromes or traumas that influence the growth and development of facial structures or body
(ii) Patients with a history of orthodontic or interceptive treatment
(iii) Patients with facial asymmetry
(iv) Patients with mixed dentition or missing teeth

CBCT was taken from the right TMJ region to minimize radiation exposure and standardize the method. The CBCT was taken with a limited field of view.

###  CBCT imaging protocol

**Figure 1:**
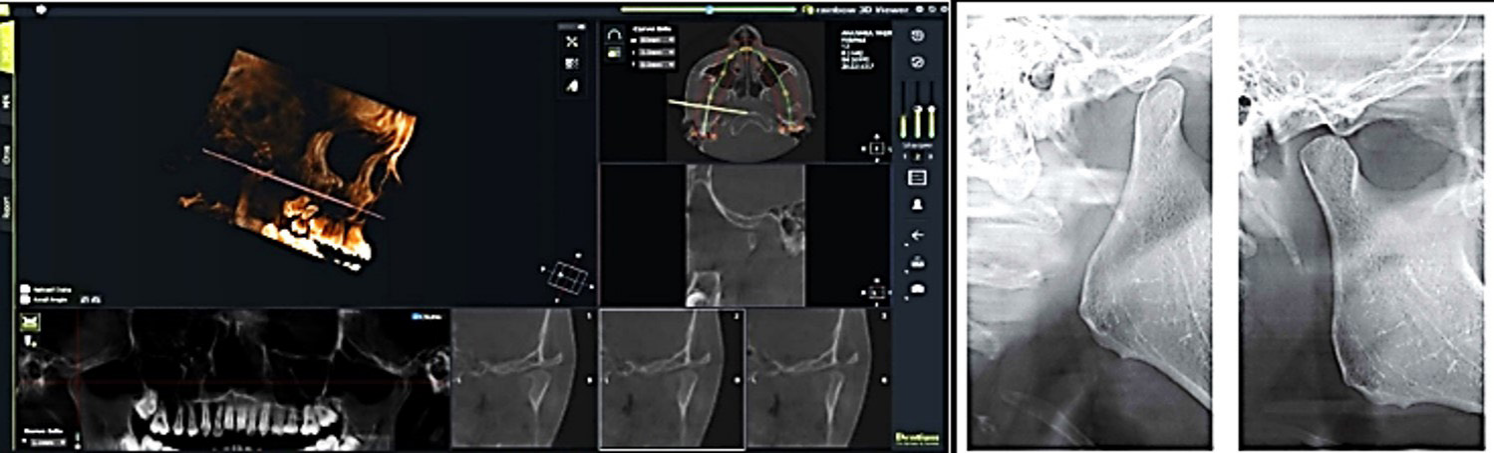
Cone-beam computed tomography (CBCT) images showing the right condyle

Figure 1 depicts the CBCT images and variables to be studied. Images were taken at 120 kV, 15 mA, and with an exposure time of 10 seconds using a CBCT machine (3D Rainbow). With the patient standing without an interocclusal separator, they were directed to keep their teeth in maximum intercuspation and refrain from swallowing or making other movements during the scanning period. The exposure setting was 110 Kv, 4 mA, 18*16 seconds scanning time. The data were in DICOM format. These data sets were uploaded into rainbow^TM^ CT software funded and sold by Dentium, South Korea, for anatomic landmark localization and TMJ measurements. All the landmarks were located on the sagittal view of the midline plane, aiming to replicate the standard procedure used in lateral cephalograms. Their positions were verified across all orthogonal planes. Rainbow software was used to assess the TMJ and its surrounding space.

 The following steps were undertaken during the study:

- Collection of diagnostic records along with pre-treatment CBCT scan
- Analysis of diagnostic records and confirmation of skeletal Class II bases with mandibular retrognathia
- Bite registration protocol
- Fabrication and delivery of the appliances
- Check-up of the patients during the period of study
- Collection and analysis of post-treatment records
- Extraction of DICOM files from the CBCT scans and importing them to a third-party software rainbow^TM^ CT for the TMJ measurements of the right-side condyle
- Comparison of TMJ measurements before and after treatment
- The parameters were recorded at baseline, 6 months, and 12 months by observing clinical variables, cephalometric variables, Arnett’s dentoskeletal factors, Arnett’s soft tissue structure, facial length, projection to true vertical line (TVL), and CBCT variables.

###  Methodology in the test group (CFJC)

####  Fabrication

**Figure 2:**
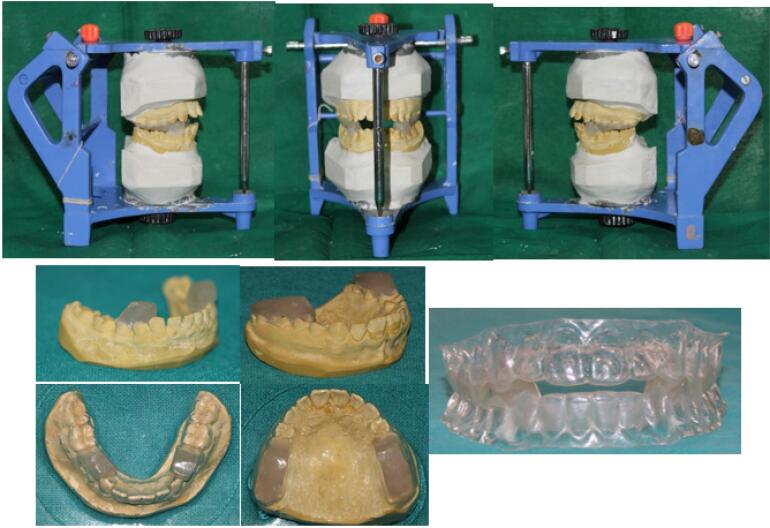
Clear functional jaw corrector appliance fabrication

 Models were mounted on an articulator with the construction bite in place. The upper bite block was angled from the mesial surface of the upper second premolar and positioned flatly over the remaining posterior teeth. The lower block was angled from the mesial surface of the lower first premolar, extending mesially to cover the premolar and, if necessary, merging into the lower incisal capping area. The inclined plane was angled at 70º to apply more horizontal force components, promoting horizontal mandibular growth. The excess thickness of each block (0.5 mm) was trimmed to accommodate the copyplast sheet. Each block was fixed to the respective cast, and a vacuum-formed procedure was carried out to fabricate the appliance. Figure 2 depicts the fabrication of the CFJC appliance.

**Figure 3:**
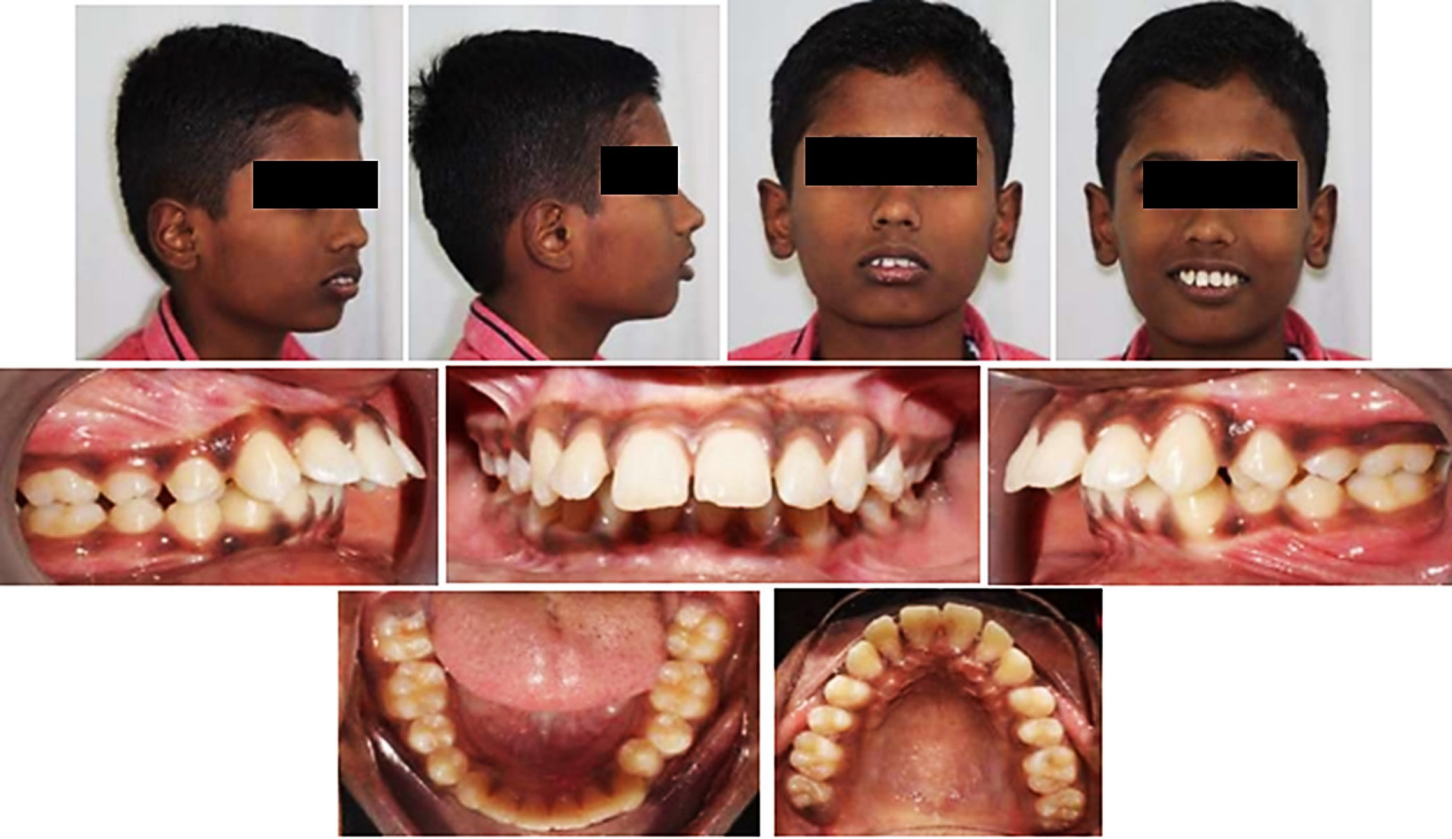
Patient’s intraoral and extraoral pre-treatment photographs of clear functional jaw corrector appliance (CFJC) (the test group)

Figure 3 depicts the preoperative extraoral and intraoral status of patients.

**Figure 4:**
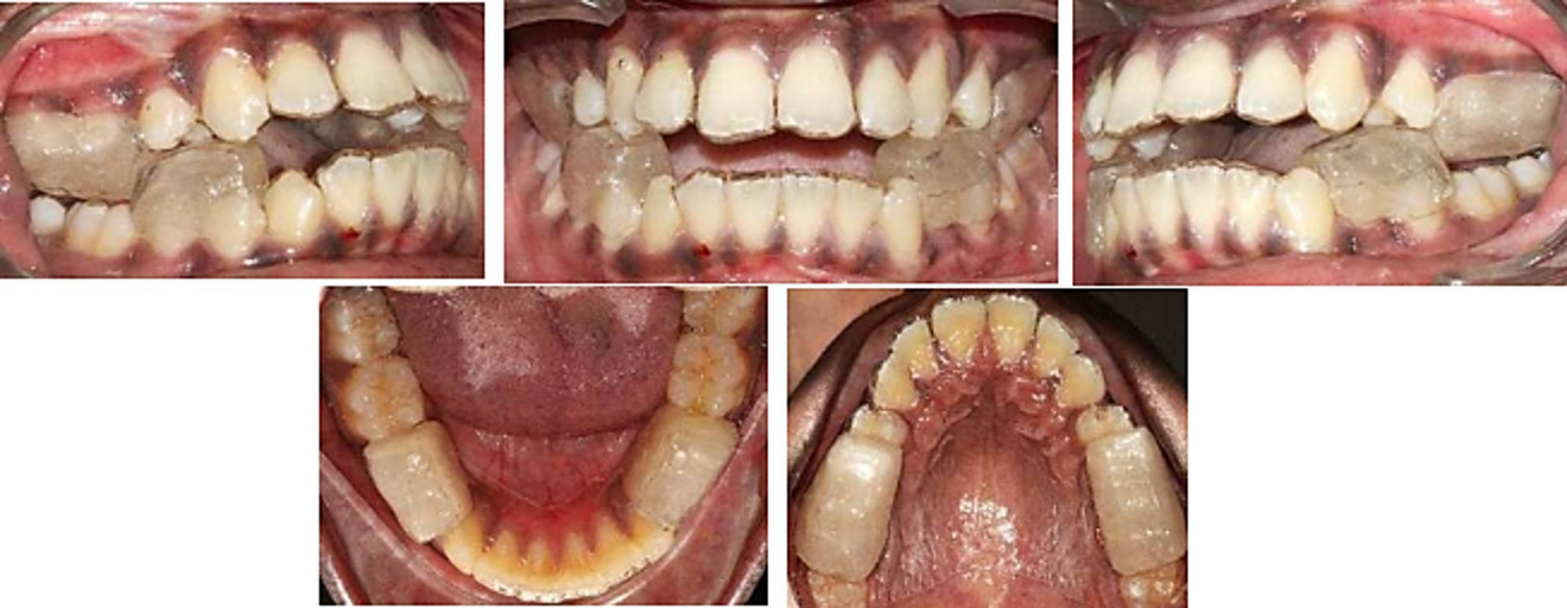
Patient’s intraoral photographs with clear functional jaw corrector (CFJC) appliance (the test group)

 Patient instructions on wear and care were provided, with a follow-up of 2 weeks for pterygoid response. Subsequent recalls at 6 weeks addressed any appliance repairs. Patients were advised to wear the appliance continuously and follow the instructions (Figure 4).

**Figure 5:**
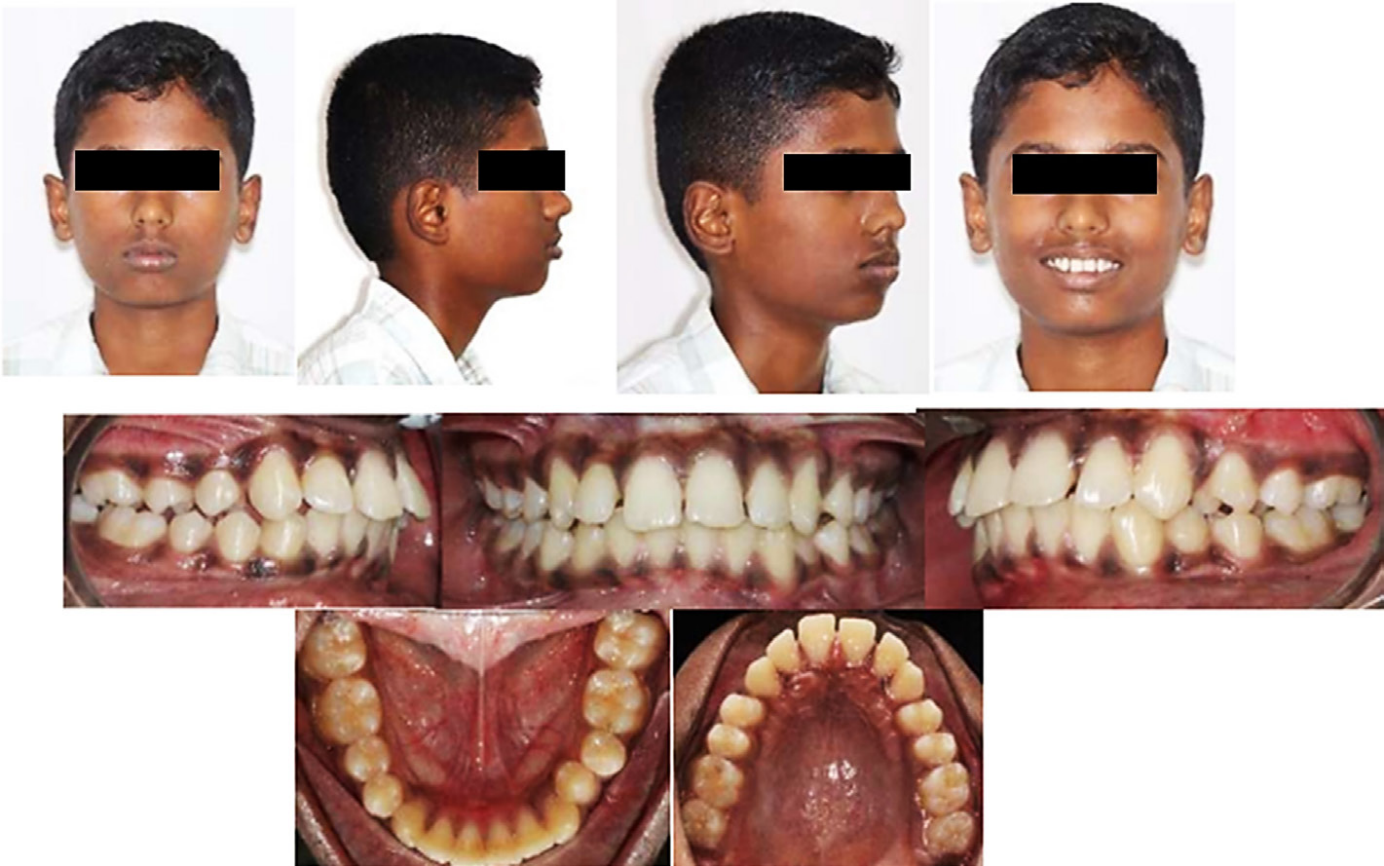
Patient’s intraoral and extraoral post-treatment photographs of clear functional jaw corrector (CFJC) appliance (the test group)

 The post-treatment extraoral and intraoral photographs of the patients are depicted in Figure 5.

###  Methodology in control group (twin block appliance)

**Figure 6:**
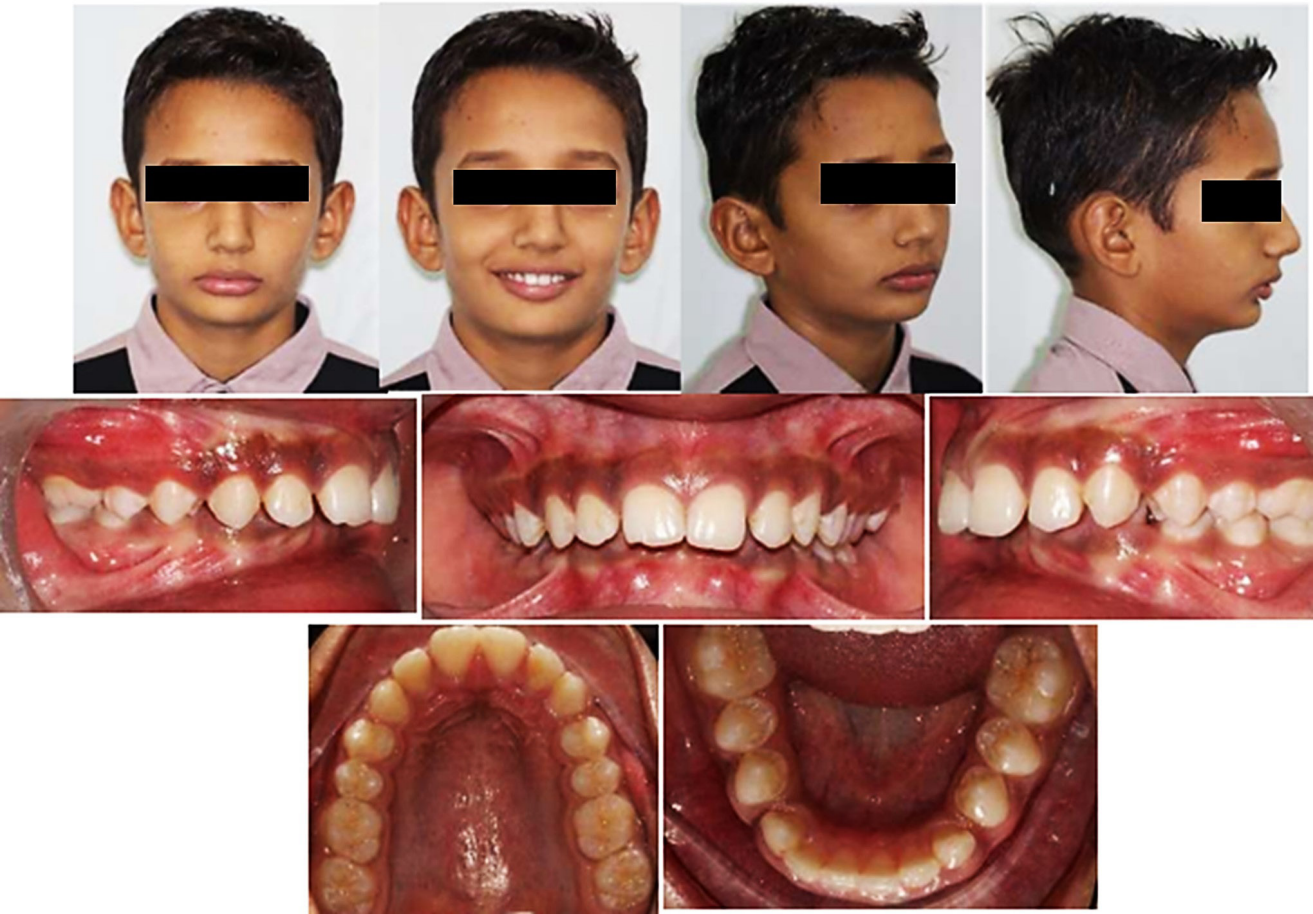
Patient’s intraoral and extraoral pre-treatment photographs of twin block appliance (the control group)

**Figure 7:**
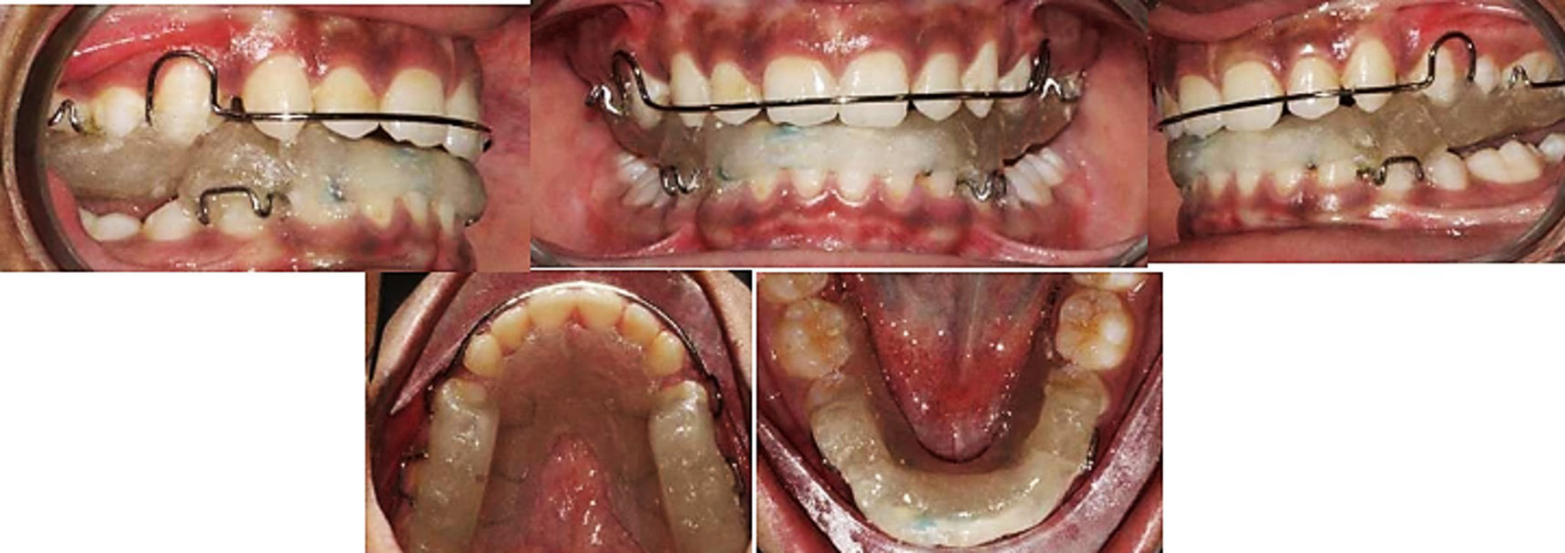
Patient’s intraoral photographs with twin block appliance (the control group)

**Figure 8:**
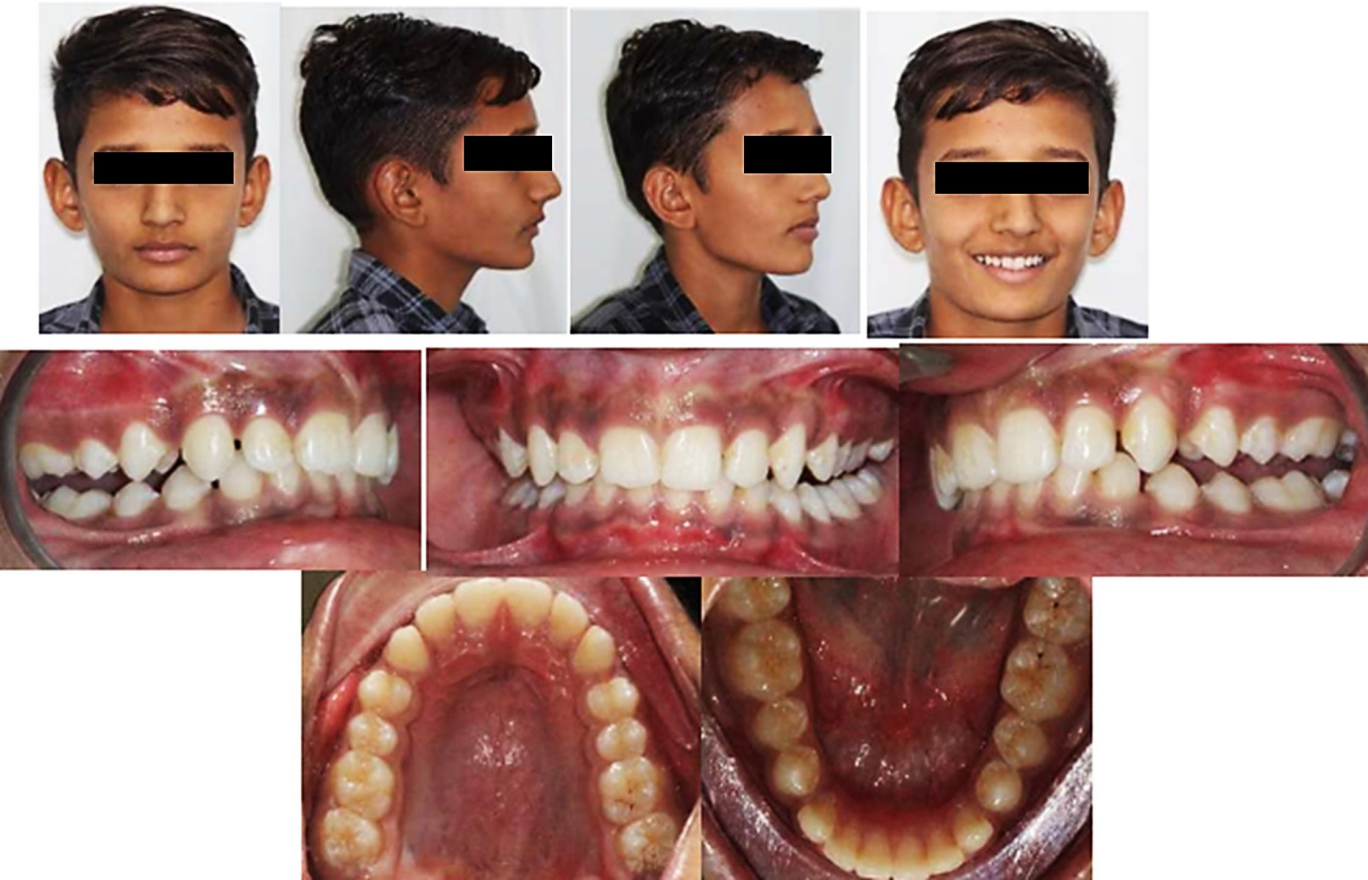
Patient’s intraoral and extraoral post-treatment photographs of twin block appliance (the control group)

Figure 6 depicts the preoperative extraoral and intraoral status of patients (control group). Patient instructions on wear and care were provided, with a follow-up of 2 weeks for discomfort assessment. Subsequent recalls at 6 weeks addressed any appliance repairs. The patients were advised to wear the appliance continuously (Figure 7), including during meals, starting with a soft diet and transitioning to normal eating gradually to adjust comfortably. The post-treatment extraoral and intraoral photographs of the patients (control group) are depicted in Figure 8.

###  Statistical analysis

 Statistical analyses were conducted using SPSS 20 (IBM SPSS Statistics Inc. Chicago, Illinois, USA). The unpaired t-test (for intergroup comparison) was used to compare quantitative data of all variables included in the study.

## Results

 The present study revealed the following outcomes when the results were compared with the baseline data between the two groups. According to Table 1, when cephalometric parameters between twin block and CFJC were compared, statistically significant differences were observed for angular measurements of SNB, ANB, and U1-NA. In contrast, the rest of the parameters did not show statistically significant differences (*P* < 0.05) in the CFJC group. Comparison of Arnett’s soft tissue parameters (Table 2) between the two groups showed significant differences in the upper lip to E-line, lower lip to E-line, upper lip protrusion, upper lip length, lower lip length, lower third of the face, maxillary exposure, and mandibular height. Additionally, notable differences were observed in the intergroup comparison of projections to TVL across all parameters.

**Table 1:** Intergroup comparison of cephalometric parameters between twin block and CFJC group

**Parameter**	**Group**	**Mean**	**Std. Deviation**	***t*** **-value**	***P*** ** value**
SNA (º)	TWIN BLOCK	-0.2000	0.76519	1.832	0.072
	CFJC	-0.5200	0.57440		
SNB (º)	TWIN BLOCK	1.2900	1.06717	-3.024	0.004
	CFJC	2.9500	2.81103		
ANB (º)	TWIN BLOCK	-1.5900	1.26691	3.186	0.002
	CFJC	-3.4133	2.86714		
Saddle angle (º)	TWIN BLOCK	-1.2667	0.46855	-1.633	0.108
	CFJC	-1.1000	0.30513		
Articular angle (º)	TWIN BLOCK	3.3067	2.56769	0.962	0.340
	CFJC	2.7333	2.01603		
Gonial angle (º)	TWIN BLOCK	2.1167	1.80021	0.177	0.860
	CFJC	2.0300	1.98705		
Y axis (º)	TWIN BLOCK	1.2567	0.94638	0.477	0.635
	CFJC	1.1133	1.34773		
(Go-Gn)-SN (º)	TWIN BLOCK	2.3000	1.20258	-.085	0.933
	CFJC	2.3300	1.51365		
U1-NA (º)	TWIN BLOCK	-1.4167	1.09987	-4.136	0.000
	CFJC	-0.3300	0.92779		
L1-NB (º)	TWIN BLOCK	0.8167	1.69423	0.475	0.636
	CFJC	0.6100	1.67422		
IMPA (º)	TWIN BLOCK	3.0000	3.37843	-0.297	0.768
	CFJC	3.8167	14.67727		
N perp pt A (mm)	TWIN BLOCK	0.2433	0.68515	1.421	0.161
	CFJC	0.0300	0.45421		
N perp Pog (mm)	TWIN BLOCK	2.0033	0.81811	-1.220	0.227
	CFJC	2.4900	2.02610		
LAFH (mm)	TWIN BLOCK	2.9367	2.07605	0.607	0.546
	CFJC	2.6533	1.49406		
Maxillary unit (Co-pt A) (mm)	TWIN BLOCK	0.1867	1.46940	1.072	0.288
	CFJC	-0.3167	2.11041		
Mandibular unit (Co- Gn) (mm)	TWIN BLOCK	3.9967	2.50688	0.702	0.486
	CFJC	3.5700	2.19139		

**Table 2:** Intergroup comparison of soft tissue parameters between twin block and CFJC group

**Parameter**	**Group**	**Mean**	**Std. Deviation**	***t*** **-value**	***P*** **-value**
U lip to E line (mm)	Twin block	0.02	0.40887	-3.089	0.003
	CFJC	0.51	0.7667		
L lip to E line (mm)	Twin block	1.6233	0.65794	-4.58	0
	CFJC	2.5533	0.89663		
Upper Lip Protrusion (mm)	Twin block	-2.4167	2.26473	-6.427	0
	CFJC	0.4733	0.96844		
Lower Lip Protrusion (mm)	Twin block	0.8933	9.06064	0.422	0.674
	CFJC	0.1933	0.56013		
Nasolabial Angle (º)	Twin block	3.7533	2.64324	-0.968	0.337
	CFJC	4.35	2.10152		
Nasion’-Menton’ (mm)	Twin block	2.4767	2.06158	0.428	0.67
	CFJC	2.3	0.92476		
Upper lip length (mm)	Twin block	-0.2633	1.51236	-4.956	0
	CFJC	1.5333	1.28636		
Interlabial gap (mm)	Twin block	-1.1033	0.68606	-0.707	0.483
	CFJC	-0.0867	7.8504		
Lower lip length (mm)	Twin block	1.64	2.75663	2.465	0.017
	CFJC	0.1167	1.96383		
Lower 1/3 of face (mm)	Twin block	0.94	1.87683	-2.704	0.009
	CFJC	1.9667	0.89533		
Mx1 exposure (mm)	Twin block	0.57	0.75208	3.939	0
	CFJC	-0.0033	0.26455		
Mandibular height (mm)	Twin block	-0.9	0.96311	-3.375	0.001
	CFJC	-0.15	0.74452		
Upper molar to PTV (mm)	Twin block	0.6767	19.0451	0.538	0.592
	CFJC	-1.21	2.39602		
Point A	Twin block	0.4	0.99516	2.93	0.005
	CFJC	-0.2067	0.54389		
Mx1	Twin block	-1.3083	0.6114	-8.053	0
	CFJC	-0.17	0.475		
Md1	Twin block	1.9933	1.73582	4.038	0
	CFJC	0.4667	1.12903		
Point B	Twin block	2.07	2.74366	-18.124	0
	CFJC	20.55	4.86428		
Pogonion	Twin block	17.1967	11.3336	-3.278	0.002
	CFJC	25.2367	7.21165		

 When CBCT parameters were compared between the two groups, statistically significant differences were observed in mean values for condylar position, condylar height, and anterior joint space (Table 3) in the CFJC group. Table 4 shows that statistically significant differences were found in the intergroup comparison of parameters assessing patient comfort and perception of the appliance, indicating better compliance with the CFJC appliance (*P* < 0.05).

**Table 3:** Intergroup comparison of CBCT parameters between twin block and CFJC group

**Parameter**	**Groups**	**Mean**	**Standard Deviation**	***t*** **-value**	***P*** ** value**
Condylar position	Twin block	0.3623	1.03777	-2.731	0.008
	CFJC	0.9850	0.69498		
Condylar height	Twin block	-0.7900	0.67696	-11.656	0.000
	CFJC	1.2003	0.64535		
Condylar width	Twin block	1.1237	1.32515	-1.496	0.140
	CFJC	1.5317	0.68949		
Posterior Joint Space	Twin block	3.6287	25.20830	0.489	0.627
	CFJC	1.3760	0.84388		
Anterior Joint Space	Twin block	-0.3633	0.70156	2.554	0.013
	CFJC	-1.0320	1.25084		
Superior condylar space	Twin block	-1.1190	0.61598	1.366	0.177
	CFJC	1.3960	0.74388		

**Table 4:** Intergroup comparison of patient comfort and perception between twin block and CFJC group

**Parameter**	**Groups**	**Mean**	**Standard Deviation**	***t*** **-value**	***P *** **value**
Pain perception	Twin block	6.3167	0.82507	11.250	0.000
	CFJC appliance	4.0833	0.70812		
Patient Comfort	Twin block	6.5167	0.51668	-2.614	0.011
	CFJC appliance	6.9500	0.74683		
Appliance appeal/ appearance	Twin block	5.3667	0.65566	-11.843	0.000
	CFJC appliance	7.3900	0.66765		
Complexity of regimen	Twin block	5.4333	0.69149	-5.705	0.000
	CFJC appliance	6.4333	0.66609		
Cost	Twin block	5.5167	0.59427	-6.517	0.000
	CFJC appliance	6.5167	0.59427		
Maintenance of oral hygiene and appliance	Twin block	5.4167	0.64438	-6.010	0.000
	CFJC appliance	6.4167	0.64438		
Visibility of appliance in the mouth	Twin block	5.5500	0.53094	13.604	0.000
	CFJC appliance	3.6000	0.57834		
Confidence	Twin block	5.4667	0.58624	-6.606	0.000
	CFJC appliance	6.4667	0.58624		
Patient perceived values for appliance	Twin block	5.5700	0.52729	-14.690	0.000
	CFJC appliance	7.5700	0.52729		
Speech related problems	Twin Block	6.3367	0.72420	10.696	.000
	CFJC Appliance	4.3367	0.72420		

## Discussion

 Class II malocclusion is prevalent among Indian populations, with rates ranging from 10% to 25%.^19^ Factors such as genetics, cultural practices, and environment influence these rates, alongside variations in diagnostic criteria and study methodologies. A higher prevalence of Class II malocclusion in boys compared to girls has been noted, highlighting a gender predilection.^20^ Early detection and appropriate orthodontic intervention are crucial in managing this significant dental issue in India. The optimal timing for myofunctional therapy initiation is debated, but studies suggest it is most effective during stages 3 to 4 of cervical vertebrae maturation (around or just after puberty).^21,22^ This study includes ages 10‒15 years for both genders, aligning with Tanner and colleagues’ findings of peak height velocity at approximately 12 years in girls and 14 years in boys.^23^

 In both groups, post-treatment changes in SNA were notable (*P* < 0.05). Group T showed a significantly greater increase in SNA compared to group C. O’Brien et al^5^ observed a nominal restraining effect on maxillary growth with the twin block appliance, constituting 13% of skeletal changes, while Illing et al^24^ reported a slight mean reduction in SNA. The forward positioning of the mandible can create a reciprocal restraining effect on the maxilla, known as the headgear effect,^25,26^ influencing maxillary growth differently in various studies.^27-31^ Maxillary position relative to the cranial base did not significantly change post-treatment in either group.

 This study showed that the decrease in ANB angle following twin block appliance therapy could result from a reduction in SNA, an increase in SNB, or both. Toth and McNamara^31^ reported a 1.8˚ decrease in ANB angle with twin block treatment, similar to findings by Illing et al.^24^ Our study also showed a significant mean reduction in ANB angle. Significant differences (*P* < 0.001) were found in N-perpendicular-to-pogonion values between the groups, indicating forward spatial changes due to mandibular anterior positioning. These results align with previous studies by O’Brien et al^5^ and Singh et al,^22^ affirming the efficacy of twin block therapy for correcting skeletal Class II malocclusions.

 The upper incisors showed reduced inclination, possibly due to a headgear-like effect from the inclined blocks. In contrast, lower incisors inclined more towards the cranial base, which was influenced by forward mandibular positioning. This contributed to decreased overjet. Studies by Clark^32^ and Illing et al^24^ demonstrated significant effects on maxillary incisor inclination with twin block therapy, emphasizing dentoalveolar correction over mandibular growth. Both study groups showed significant mandibular length increases (*P* < 0.001), with comparable skeletal effects between appliances and minor dental differences, as supported by previous research.^24,33^

 The soft tissue cephalometric analysis serves as a critical method for both vertical and horizontal profile assessment, extending the principles outlined in “Facial Keys to Orthodontic Diagnosis and Treatment Planning” by Arnett.^34^ Quintão et al^35^ noted significant upper lip changes due to maxillary incisor retroclination post-functional appliance treatment, contrasting with Morris and colleagues’^36^ findings of no significant sagittal upper lip change despite reduced overjet. In our study, both appliance groups showed decreased upper lip projection. Lower lip changes were significant in both groups, with greater advancement observed in the CFJC appliance group. Nasolabial angle changes were not significant, consistent with recent meta-analyses.^37^

 The present study evaluated condylar position changes after treatment with twin block and CFJC appliances. CFJC group showed more significant shifts, consistent with prior research by Arumugam et al^38^ Condylar movements included anterior shifts, akin to findings with Herbst appliances,^39^ twin block,^40-42^ and other functional appliances.^43^ Condylar height increased notably in both groups, contrasting with decreased heights in untreated controls.^44^ Condylar width increased, more so in CFJC, aligning with findings by Parvathy et al.^45^ Condyle growth in functional therapy enhances mandibular length and volume,^45,46^ promoting sagittal and vertical condylar dimensions. TMJ changes noted anterior and posterior joint space adjustments after treatment, similar to findings by Yildirim et al^47^ and Bayram et al.^48^ Functional appliances influence articular fossa growth, aiding mandibular repositioning.^49^ Despite challenges in assessing fossa remodeling, TMJ space alterations indicate treatment efficacy. The study’s comprehensive analysis supports CFJC’s superiority in achieving favorable skeletal, dental, and soft tissue outcomes in Class II malocclusion over twin block within a 12-month treatment period.

 Patient compliance and satisfaction were assessed for twin block and CFJC appliances based on pain perception, comfort, appearance, regimen complexity, cost, hygiene, visibility, confidence, and speech issues. Both groups showed significant differences. Twin block was associated with higher pain perception, visibility, and speech-related problems, while CFJC was associated with comfort, appearance, regimen simplicity, cost-effectiveness, patient confidence, and perceived appliance value. Similar findings in patient satisfaction were reported by Golfeshan et al,^50^ highlighting reduced speech issues with clear aligners. Oliver and Knapman’s study^51^ and Thirumurthi and colleagues’^52^ psychological assessments also underscored patient satisfaction and challenges associated with orthodontic treatment, aligning with our study’s outcomes.

## Conclusion

 This prospective clinical study aimed to evaluate condylar position changes using CBCT in treating Class II malocclusion with the twin block and CFJC appliances. Key findings include:

1. Both appliances significantly improved skeletal, dental, and soft tissue parameters post-treatment.
2. The CFJC appliance yielded superior skeletal, dental, and soft tissue changes compared to the traditional twin block.
3. TMJ changes were observed in both groups, with the CFJC group exhibiting more pronounced changes than the twin block group.
4. CFJC showed enhanced efficacy due to more significant condylar remodeling compared to the twin block appliance.
5. Patient compliance was higher in the CFJC group, possibly due to reduced treatment duration compared to traditional twin block therapy.

These results highlight that the CFJC appliance emerges as a preferred treatment for Class II malocclusion, demonstrating superior efficacy in enhancing skeletal, dental, and soft tissue changes, as well as promoting condylar remodeling and improved patient compliance.

## Competing Interests

 The authors declare no competing interests related to the publication of this work.

## Ethical Approval

 This study received ethical approval from institutional review board (PIMS/DR/PhD/RDC/2022/134).
